# Unravelling the case of suspected ectopic ureter in a young adult patient

**DOI:** 10.1016/j.radcr.2024.11.086

**Published:** 2025-01-02

**Authors:** Leni Santiani, Tjahjodjati, Basofi Sukiman

**Affiliations:** a Department of Radiology, Hasan Sadikin Academic Medical Center-Faculty of Medicine, University of Padjadjaran, Jatinangor, Indonesia; b Department of Urology, Hasan Sadikin Academic Medical Center-Faculty of Medicine, University of Padjadjaran, Jatinangor, Indonesia

**Keywords:** Ectopic ureter, Incontinence, Magnetic resonance urography

## Abstract

An ectopic ureter (EU) opens outside the bladder's trigone, a rare condition with an incidence of 0.05%-0.025%. It often causes continuous urine leakage and frequent urination, particularly in females. Diagnosing EU is challenging, as conventional radiologic techniques often fail to identify it accurately. Excretory urography and magnetic resonance urography are more effective in visualizing the ectopic insertion of the ureter. This study reports a rare case of suspected ectopic ureter in an adult and reviews the radiologic diagnostic approach. A 26-year-old female presented with lifelong incontinence. She reported constant dribbling and required frequent pad changes. Physical examination was normal, and she had no fever or pain. Imaging revealed a dilated and tortuous right ureter inserting into the vagina, indicating a suspected ectopic ureter. Ectopic ureter is a rare congenital anomaly of the urinary system, often associated with other anomalies or syndromes. It commonly presents with incontinence, but symptoms can vary from asymptomatic to renal failure. Radiologic examination is crucial for diagnosing this condition.

## Introduction

The ureters are muscular tubes that transport urine from the kidneys to the bladder, developing from the ureteric bud during embryonic development [1,2]. An ectopic ureter is a rare condition where the ureter opens at a location other than the bladder's trigone, with an incidence rate of 0.05%-0.025% [3]. It often causes continuous urine leakage, particularly in females, and usually originates from the upper renal pole of a duplex system [4]. While rare in adults, ectopic ureter should be considered in cases of lifelong incontinence [5]. Diagnosis is challenging, as conventional radiologic methods often fail to identify the condition, though excretory urography and magnetic resonance urography are useful for visualization [6,7]. This study focuses on a rare adult case of suspected ectopic ureter and the diagnostic radiologic approach.

## Case presentation

**Fig. 1:**
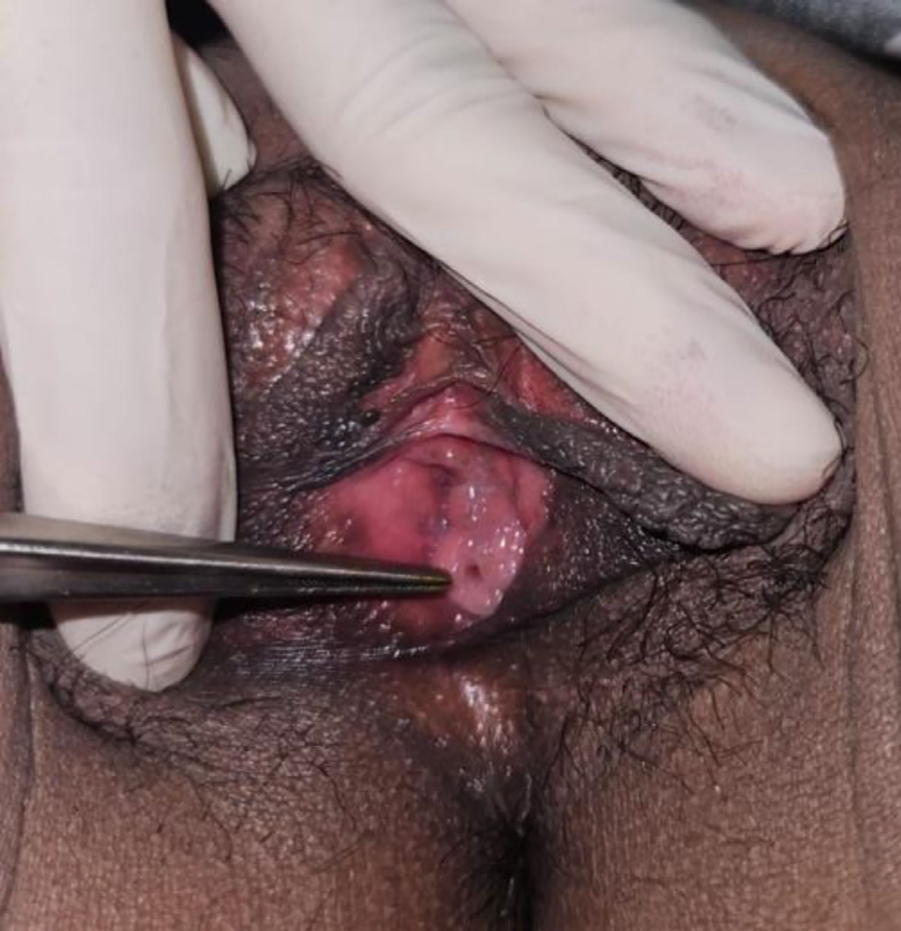
Vaginal clinical findings.

**Fig. 2:**
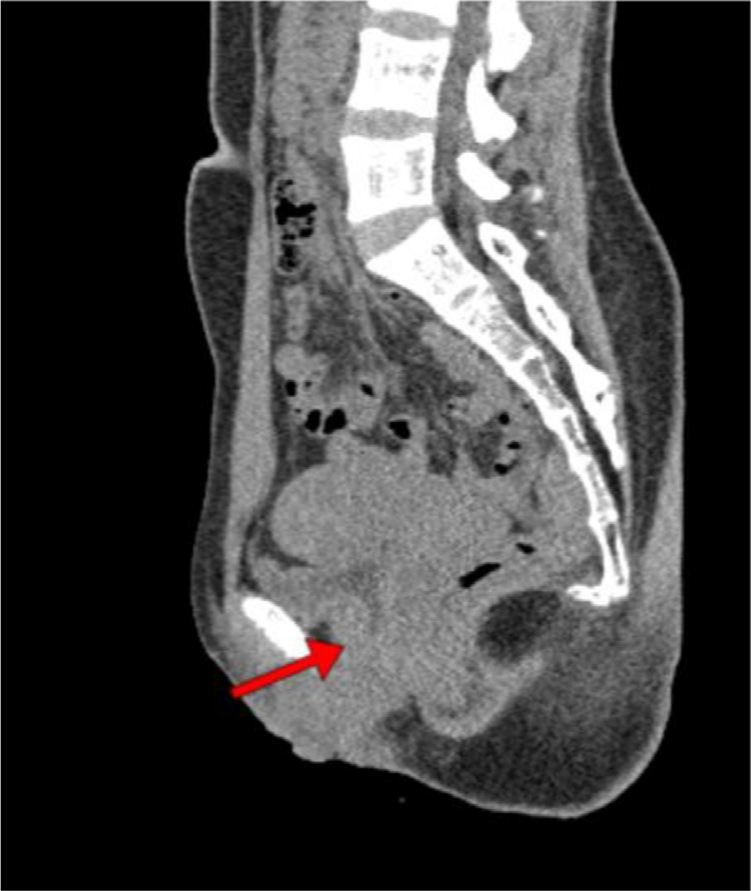
Noncontrast abdominal CT scan.

**Fig. 3:**
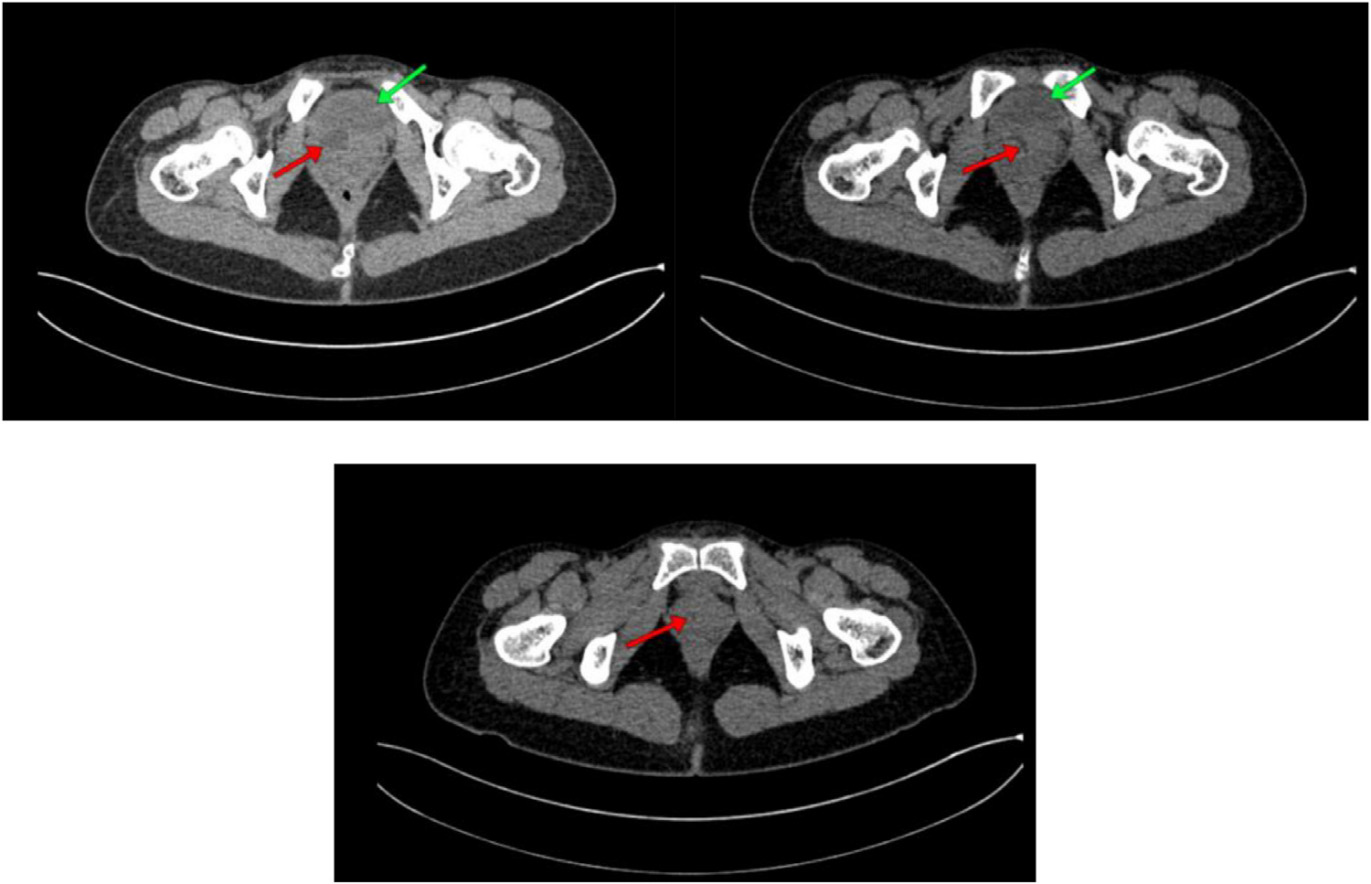
Noncontrast abdominal CT scan. Green arrow: bladder; red arrow: dilated and tortuous ureter still running below the urinary bladder.

**Fig. 4:**
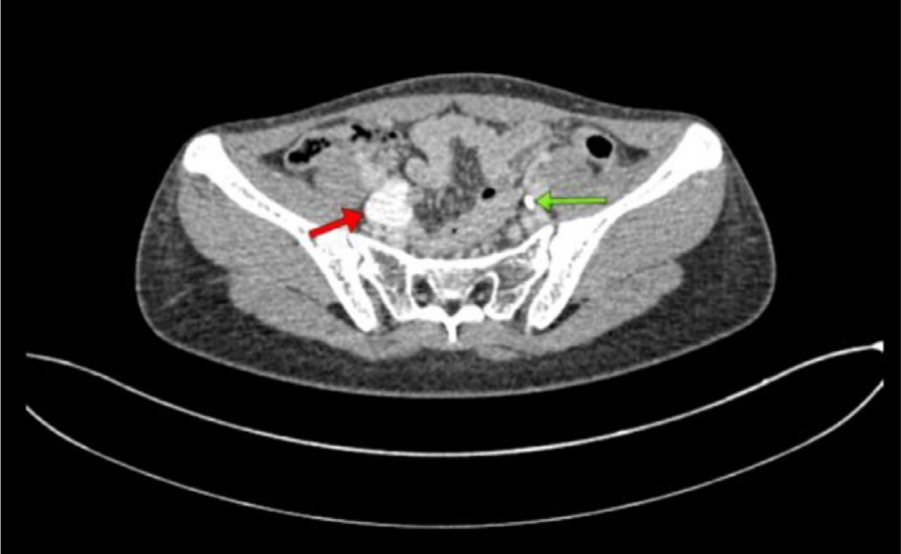
Contrast-enhanced abdominal CT scan: Dilated right ureter (hydroureter) with tortuous.

**Fig. 5:**
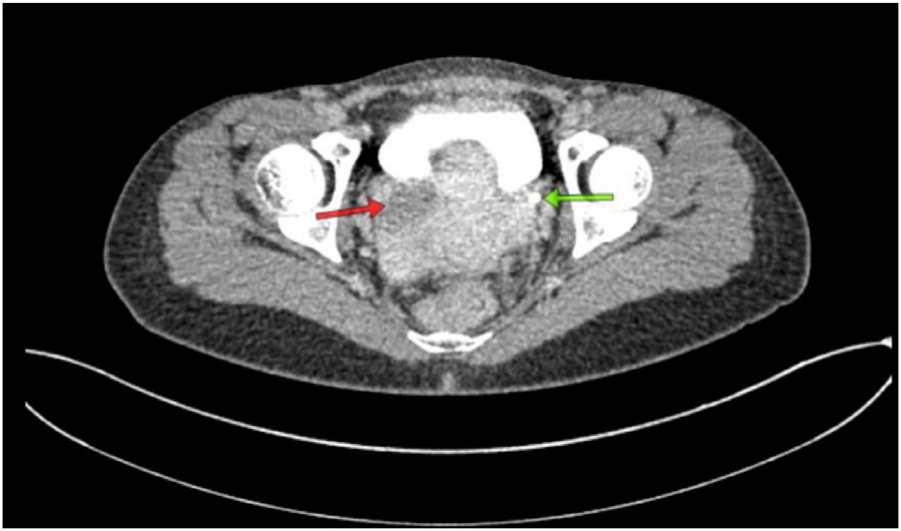
Contrast-enhanced abdominal CT scan: The contrast does not appear to fill the entire right ureter due to the size of the right ureter which is dilated (dilated/hydroureter) and tortuous, while the left ureter appears to have contrast passing through and not flowing into the urinary bladder.

**Fig. 6:**
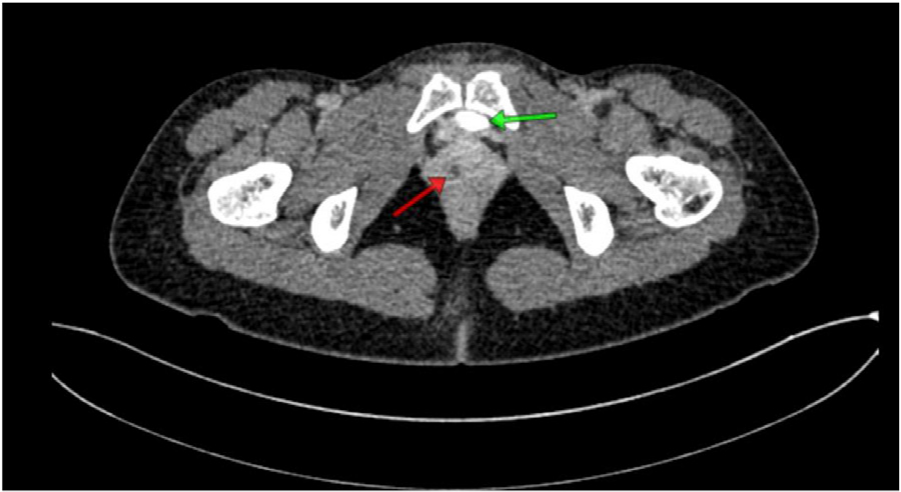
Contrast-enhanced abdominal CT scan: The urinary bladder (green arrow) and the distal right ureter are seen opening into the vagina (red arrow).

**Fig. 7:**
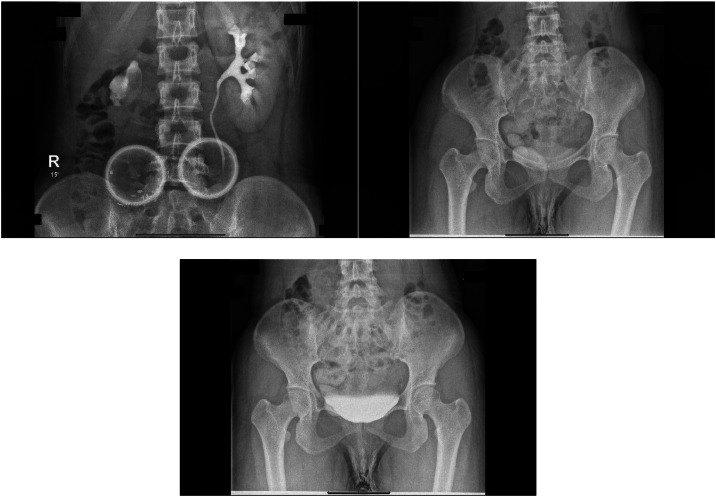
BNO-IVP.

**Fig. 8:**
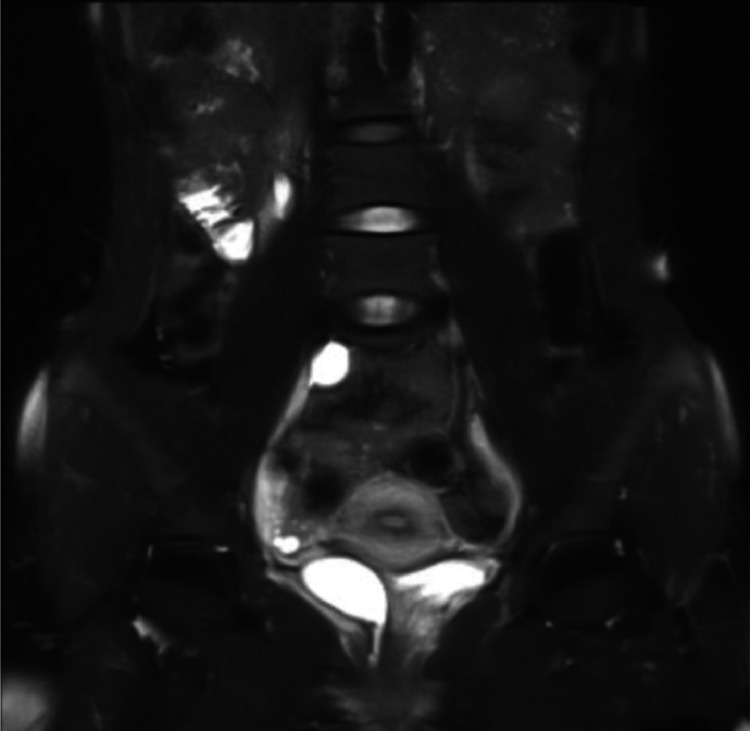
MRI T2 sequence coronal.

**Fig. 9:**
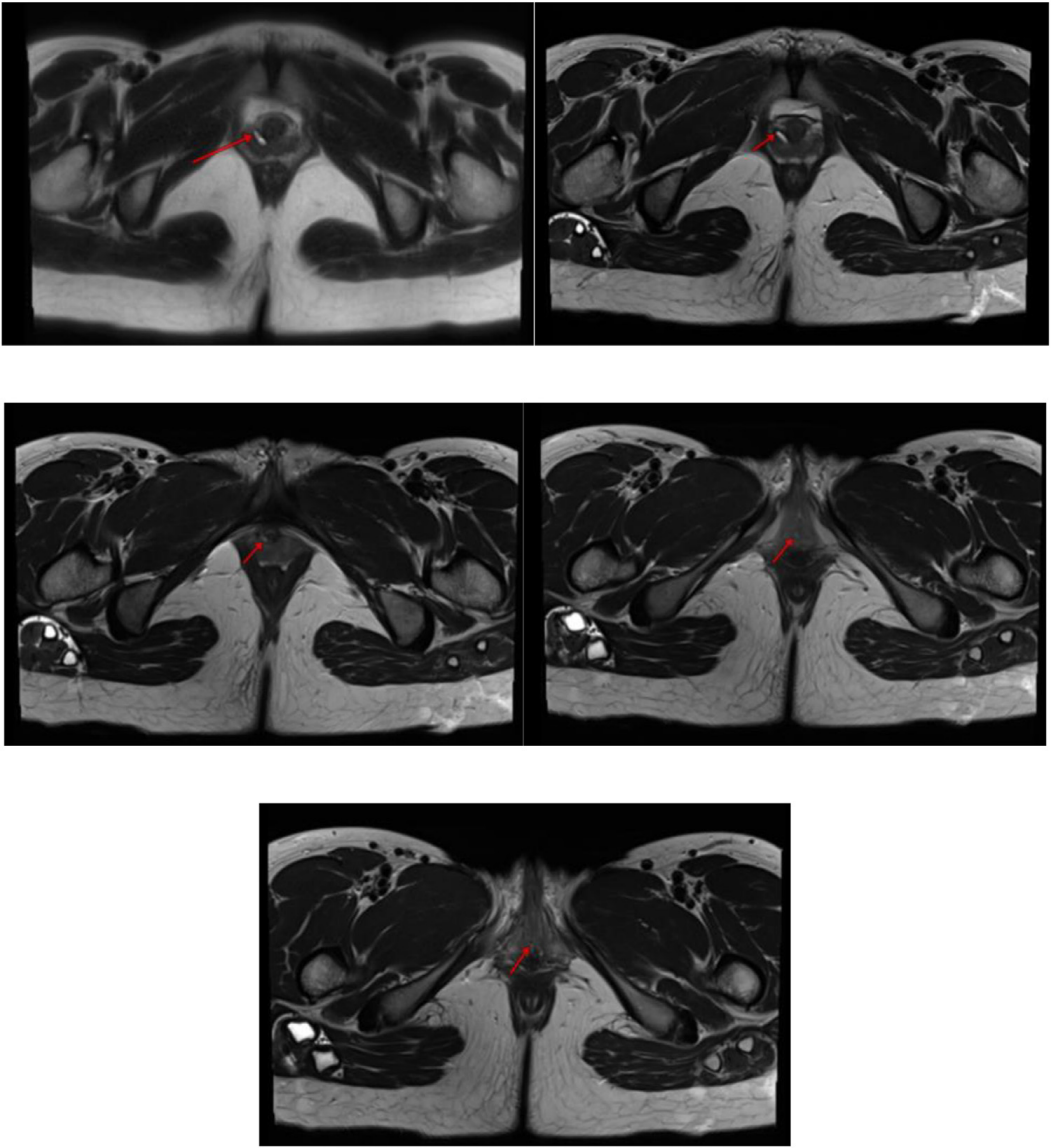
MRI T2 sequence.

**Fig. 10:**
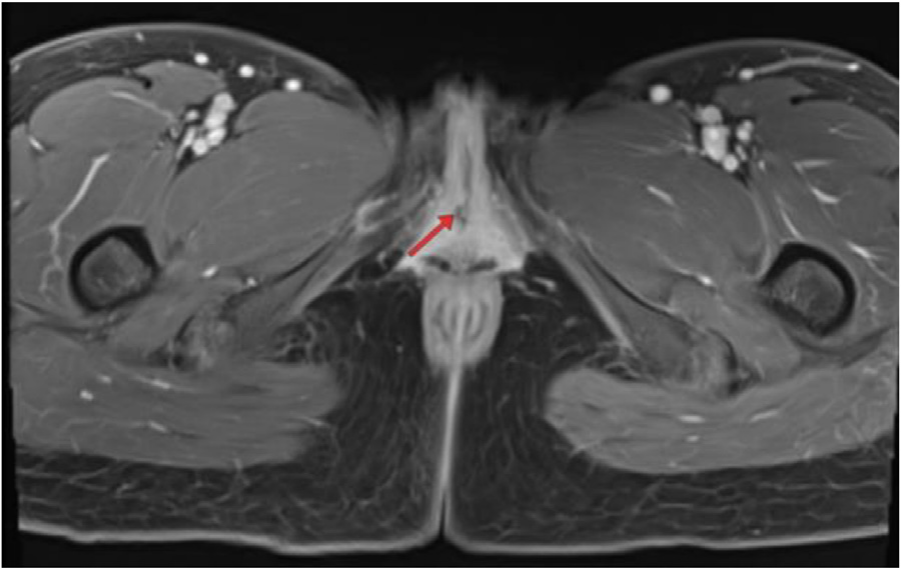
MRI T1 FS sequence.

A 26-years old female patient presented with complaints of incontinence since birth. She reported yellow clear or dark yellow fluid discharge from her genital area, varying with her fluid intake. She experienced dribbling during urination. The patient changed her pads twice a day due to constant wetness from urine. She did not report any fever or pain. Physical examination showed vital signs within normal limits and no masses were found in the abdomen. The patient is suspected of having true incontinence with a possible cause of an ectopic ureter Fig. 1, Fig. 2, Fig. 3, Fig. 4, Fig. 5, Fig. 6, Fig. 7, Fig. 8, Fig. 9, Fig. 10.

The results of the abdominal CT scan showed dilation of the right renal pelvis system, with dilation and tortuosity observed from the proximal to distal ureter. The distal ureter appeared to insert into the vagina. The CT scan impression is a suspected ectopic ureter with distal ureter insertion into the vagina and right renal pelvis ectasis.

The BNO-IVP examination showed that the left kidney appeared normal. The right kidney showed contrast still filling the renal pelvis area. During voiding, the right ureter opening was not visible. When the contrast fully filled the urinary bladder, it did not fill the right ureter lumen distally due to hydroureter and tortuosity of the right ureter. The MRI examination showed that on the T2 sequence coronal slice, the distal right ureter became progressively narrower and did not insert into the urinary bladder. The ureter end appeared to insert in the vaginal vestibule between the urethra and the introitus. The MRI impression was an ectopic ureter with suspected distal ureter insertion in the vaginal vestibule or distal vagina. Grade II right hydronephrosis was accompanied by right hydroureter.

## Discussion

Ectopic ureters are uncommon cases especially in adult. According to the Weigert-Meyer law, the upper segment ureter of a duplex kidney is typically involved. The orifice of an ectopic ureter can be found not only in the bladder but also in various parts of the genitourinary system, including the vagina. During embryonic development, the lower pole of the ureteric bud separates earlier and migrates superiorly and laterally, while the urogenital sinus moves cranially to become the upper pole [8,9].

It may take some time to diagnose them in people who show no symptoms. Mostly, patient will presented with incontinence since birth. However, when a patient exhibits no symptoms, a high degree of suspicion is required to make the diagnosis. Ectopic ureter incidence is extremely low, ranging from 0.05% to 0.025 percent [3].

The differential diagnosis of ectopic ureter involves distinguishing it from other conditions that can cause similar symptoms, such as continuous urinary incontinence or recurrent urinary tract infections. Common differential diagnoses include vesicoureteral reflux (VUR), neurogenic bladder dysfunction, and persistent vesicovaginal fistula [10]. Diagnostic imaging like excretory urography, magnetic resonance urography, and cystoscopy can help rule out these conditions by providing clearer visualization of the urinary tract and the ectopic ureter's insertion point [11,12]. For diagnosis in circumstances where other modalities are not conclusive, magnetic resonance imaging and magnetic resonance urography has higher soft tissue contrast and resolution [13,14].

The ectopic ureter can be linked to a number of anomalies, including ureterocele, duplex kidneys, ureter duplication, and syndromic associations like Zinner syndrome and VACTERL syndrome (vertebral defects, anal atresia, cardiac defects, tracheo-oesophageal fistulas, renal anomalies, and limb abnormalities) [14,15]. According to the study by Choudhury et al., duplex collecting systems with an ectopic ureter occur in about 30% of instances, with the other cases having a single system with an ectopic ureter. Of the patients in Rani et al study, 83.4% had a single collecting system and 16.6% had a duplex collecting system. Furthermore, our investigation revealed no instances of bilateral involvement; 83.4% of cases had isolated left system participation and 16.6% had isolated right system involvement [14,16].

## Conclusion

Ectopic ureter is a rare congenital anomaly of urinary system. This condition is associated with other anomalies or syndrome. Patients with ectopic ureter mostly presenting with incontinence, but it can range from asymptomatic to renal failure. Radiology examination is an important diagnostic workup for diagnosing ectopic ureter.

## Patient consent

Written informed consent for publication of their case was obtained from our patient.

## Ethical consent

Written informed consent was obtained from the patient.

## CRediT authorship contribution statement

**Leni Santiani:** . **Tjahjodjati:** . **Basofi Sukiman:** Writing – original draft, Writing – review & editing, Investigation.
